# The Curability of Insanity

**Published:** 1887-03

**Authors:** 


**The Curability of Insanity.** A Series of Studies by PLINY EARLE, A. M., M. D., late Superintendent of the State Lunatic Hospital at Northampton, Mass.

To Dr. Pliny Earle, as much perhaps as to any other writer, is due the credit for the accepted ideas as to the curability of insanity. It is ten years since his first articles were published on this subject, and it certainly is not presumptious for him to claim that they have greatly modified the aspect of insanity. The statistics which he presents, as well as the arguments, lead necessarily to the conclusion that it is a great saving to the state to maintain hospitals for the cure of the insane, and that it is a curable disease.

